# Multimodal tele-epileptology: Challenges on the way to interoperable medical data

**DOI:** 10.1016/j.cnp.2025.02.004

**Published:** 2025-02-28

**Authors:** Sigrid Mues, Arndt Ebert, Marc Kämmerer, Marcus Kremers, Ulrich Sliwka, Rüdiger Hilker-Roggendorf, Dirk Woitalla, Iris Adelt, Thomas Günnewig, Ana Miron, Sulev Haldre, Tipakorn Tumnark, Kanjana Unnwongse, Wenke Grönheit, Tim Wehner, Vanessa Behrens, Jörg Wellmer

**Affiliations:** a Ruhr-Epileptology, Department of Neurology, University Hospital Knappschaftskrankenhaus, Ruhr-University Bochum 44892 Bochum, Germany; b BESA GmbH, 82166 Gräfelfing, Germany; c VISUS Health IT GmbH a Company of CompuGroup Medical SE & Co. KGaA, 44801 Bochum, Germany; d MedEcon Ruhr GmbH, 44801 Bochum, Germany; e Neurologische Klinik, Sana-Klinikum Remscheid 42859 Remscheid, Germany; f Klinik für Neurologie, Klinikum Vest, 45657 Recklinghausen, Germany; g Klinik für Neurologie, St. Josef-Krankenhaus Kupferdreh, 45257 Essen, Germany; h Neurologie, St. Marien Hospital Lünen 44534 Lünen, Germany; i Abteilung Geriatrie/Neurologie, Elisabeth Krankenhaus Recklinghausen 45661 Recklinghausen, Germany; j North-East Carpathian Epilepsy Center, Suceava, Romania; k Neurology Clinic, Tartu University Hospital, Tartu, Estonia; l Neurolgy Department, Neurological Institute of Thailand, Bangkok, Thailand

**Keywords:** Telemedicine, Interoperability, Data exchange, Epileptology, Consultation, DICOM

## Abstract

**Objective:**

To realize multi-modal data exchange for telemedicine in epilepsy.

**Methods:**

TE Ruhr is a multicenter, prospective pilot study. Primary endpoint of the study was the technical implementation of a platform between an epilepsy center and regional neurological departments and international cooperating epilepsy centers, respectively. A multi-professional board was established to develop technical workflows. After completion of the study a survey was conducted among users of the regional arm.

**Results:**

Two workflows were developed, 1) a combination of web-application and use of an established teleradiology network, and 2) a web-application only data exchange. Technical workflow 1 comprised local EEG conversion into to a standard format (.besa) and its shipping as DICOM RAW object. Technically, both workflows could be implemented. Yet, workflow 1 was not realisable in peripheral hospitals. Via workflow 2, 149 consults for 144 patients were completed. Users of the regional arm were satisfied (1.6 on a grading scale of 1–6 (1-very good, 6- very bad)).

**Conclusion:**

Technical feasibility alone does not determine the actual use of telemedicine. Web applications enables multimodal data exchange, but usability is limited due to lack of interoperability.

**Significance:**

Genuine interoperability of medical data remains the desired goal for multi modal data exchange.

## Highlights

• We developed technical workflows for multi-modal data exchange in tele-epileptology.
• Web applications enable multimodal data exchange.
• Genuine interoperability of medical data remains the desired goal.

## 1 Introduction

Telemedicine is the delivery of health care services across spatial distance by using information and communication technologies (World Health Organization, 2021). According to the WHO, telemedicine divides into consultations between remote client and healthcare provider (teletherapy), transmission of health, diagnostic or monitoring data (telemonitoring) and consultations between health care providers (telecooperation) *(*World Health Organization, 2023). Telecooperation, which usually takes the form of teleconsultations, primarily enables better availability of specialist knowledge. The clinical added value of telecooperation is increasingly being recognized by health insurers through the introduction of operating and reimbursement rules. In the current procedural terminology code of the American medical association, the digits 99446–99449 generally code for consultations between service providers (Gross et al., 2020). In Germany, there are separate procedure keys for telemedically supported intensive care and stroke treatment *(*Bundesinstitut für Arzneimittel und Medizinprodukte, 2024, Bundesausschuss, 2023*)*.

Epileptology is a neurological subspecialty that addresses the differential diagnoses of seizure- disorders (including epileptic and non-epileptic psychogenic seizures, syncopes and other causes of transient loss of consciousness), emergency and elective treatment of epileptic seizures and status epilepticus, treatment of patients in special conditions such as pregnancy or in the setting of comorbidities. Also, presurgical assessment including non-invasive and invasive video-EEG and multimodal image guided epilepsy surgery belong to the spectrum of specialized epilepsy units. Telemedicine in epileptology (tele-epileptology, TE^1^) therefore should be able to handle all data formats being used in this field (Mues et al., 2021). Amongst these the most important ones are structured clinical data, MRIs, (video -) EEG and (smart phone) videos of seizure-like events.

Well-established commercially available solutions exist for the transfer of imaging data, thanks to the use of the DICOM (digital imaging and communications in medicine^2^) standard (National Electrical Manufacturers Association, 2023). The exchange of structured and unstructured data (report forms, laboratory values, doctors’ letters), is hampered by the lack of interoperability between systems. There is also no standardized solution yet for the integration of (smart phone) videos into clinical workplace systems which comply with current data security and data protection requirements as demanded by the European General Data Protection Regulation (GDPR)^3^ (Rammé et al., 2021).

**Fig. 1:**
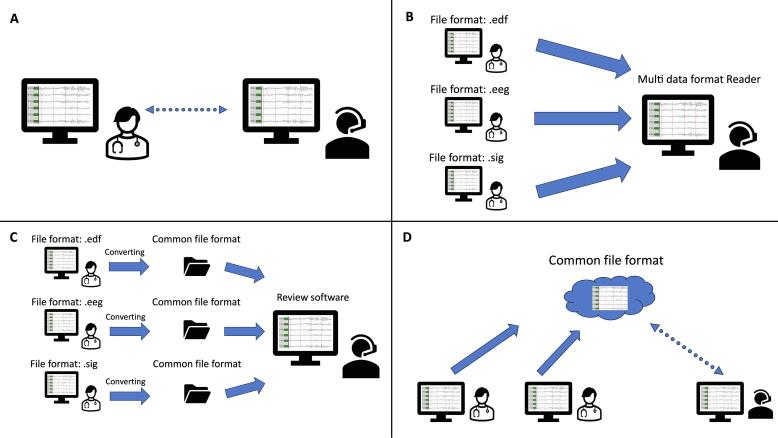
Telemedical EEG data solutions: A: Remote access to EEG data, no data transfer (dotted arrow) B: EEG transfer in proprietary format and use of a multi data format reader C: Converting EEG into a common format before EEG transfer D: Cloud Service for EEG storage.

The major challenge for TE is the transfer of (video-)EEG files, mainly due to a missing unified data format. Nearly every manufacturer provides its own proprietary format. To overcome this problem, different potential solutions are conceivable (see Fig. 1, Table 1.).

**Table 1:** Advantages and Disadvantages of different telemedical EEG data solutions.

Solution	Advantage	Disadvantage
A: Remote access to EEG data	• data stays on side, no risks of data transfer • EEG data can be read by the proprietary software	• consultation usually has to be performed as real-time (synchronous) telemedicine, this is a disadvantage when evaluation of data is time consuming
B: EEG transfer and use of a multi data format reader	• asynchronous consultation possible • no further software installation on the sending side	• risks of data transfer • limitations of multi data format reader (e.g. check of data integrity, annotations missing, no synchronization of video and EEG)
C: Converting EEG into a common format before EEG transfer	• asynchronous consultation possible • only one review software on receiving side • data integrity is guaranteed when using a predefined standard format	• risks of data transfer • need for converter software installation on the sending side
D: Cloud Service for EEG storage	• location and device-independent access to the data	• large amounts of storage capacities required • Data security concerns

Being aware of these challenges, we carried out a pilot project with industry partners from 2018 to 2022 to establish regional and international tele-epileptology. We present successful and unsuccessful workflows and discuss ongoing technical limitations.

## 2 Methods

Ruhr Epileptology is a certified epilepsy center (Level 4 according to NAEC *(*National Association of Epilepsy Centers, 2024)) at University Hospital Knappschaftskrankenhaus Bochum, Germany (UKB^4^). It is the academic epileptological reference center for the western German Ruhr Metropolitan Region with >5 Mio inhabitants. It offers all above mentioned elective and emergency epilepsy and non-epileptic seizure associated services to neurological departments and neurologists in private office in the region. It also cooperates with international epilepsy centers, particularly for epilepsy surgery issues.

The “Pilot Project Tele-Epileptology Ruhr” (TE Ruhr^5^) intended to digitize pre-existing co-operations, facilitate and accelerate the exchange of patient related data including (video-) EEG data, thereby optimizing patient treatment on site or preparing patient transfer to the epilepsy center. The project was designed with the needs of regional and international partners in mind.

A multi-professional board was established to discuss and prepare the implementation. Two guiding strategies were agreed upon:

1. Physicians at Ruhr-Epileptology were asked to formulate specific requirements for patient related datasets necessary for responding to comprehensive tele-epileptological consultations. This ensured that the clinical-medical perspective was the top priority in this project and that the technical developments were geared towards this.
2. Preexisting and clinically established telemedicine networks should be integrated into the newly designed TE Ruhr concept (e.g. opening teleradiology applications to epileptological formats and contents).

A technical development cooperation was agreed upon with the companies BESA GmbH, Gräfeling, VISUS Health IT GmbH, Bochum and MedEcon Telemedizin GmbH, Bochum (all Germany) in collaboration with Knappschaftkliniken Solution GmbH as the IT unit of UKB. For the respective tasks in the project: see results. Panvision GmbH, Essen, was commissioned to develop a web application.

Medical cooperation partners were regional and international neurological departments which had already referred patients to Ruhr Epileptology in the run-up of the project. In each department, liaison physicians were appointed to state their medical and technical expectations on the project, to later discuss patients with the specialists at Ruhr-Epileptology, and to give continuous feedback with regards to functionality of the tool. Training sessions were held at the start of the project and throughout.

The principles of the European GDPR were included into the project. In addition, national German requirements with regard to patient information and consent were taken into account. Study design, data flow and graphical user interface design were approved by the data security management of UKB.

The study is a multicenter, prospective pilot study which ran over 46 months (09/2018–06/2022). It has received a positive vote from the Ethics Committee of the Medical Faculty of the Ruhr University Bochum (Rg.-Nr. 17-3617) and was funded by a grant of the Werner Richard – Dr. Carl Dörken foundation, Herdecke, Germany.

Primary endpoint of the study was the technical implementation of a tele-epileptological platform between regional neurological departments not specialized in epilepsy and the Ruhr-Epileptology (regional arm TE Ruhr) and between international cooperating epilepsy centers and the Ruhr-Epileptology (international arm TE Ruhr), respectively. Since 2018 five, and from 2020 eight regional, non-university, neurological departments participated in the regional arm of TE Ruhr. In the international arm, we cooperated since 2018 with three international epilepsy centers. After completion of the study 06/2022, a pseudonymized online survey was conducted among users of the regional arm to evaluate the tele-epileptological consultation service (until 11/2022) (secondary endpoint).

## 3 Results

### 3.1 Agreement on content and specifications

Medical information that should be able to be provided via TE Ruhr according to the consensus of epileptologists at Ruhr-Epileptology contain all data provided in Table 2.

**Table 2:** List of all medical and technical information that should be communicable with TE Ruhr partner departments.

**Data category**	**Content**
Clinical data	Structured epileptological history:
	• Age at onset of seizure disorder • Classification and etiology of epilepsy • Classification of seizures • Differential diagnosis taken into consideration • Epilepsy-predisposing factors • Family history • Seizure semiology • Results of EEG and MRI • Current and previous medications
Videos	(Smartphone-) videos of seizures recorded by patients or third persons
Neurophysiological data	(Video)-EEG (Formats: e.g..eeg,.edf,.sig)
Imaging data	MRI, CT, PET-CT
Documents	PDF, Microsoft office formats, jpeg
Consultation request	• Diagnostic confirmation of epileptic or non-epileptic seizures • Recommendation for further diagnostic work-up • Request to perform special examinations as a service (e.g. MRI or EEG post-processing) • Given indication for presurgical work-up? • Request for therapeutic recommendation (including special medical conditions such as pregnancy or comorbidity) • Request for admission to Ruhr-Epileptology • Sociomedical counseling

### 3.2 Technical realisation

**Fig. 2:**
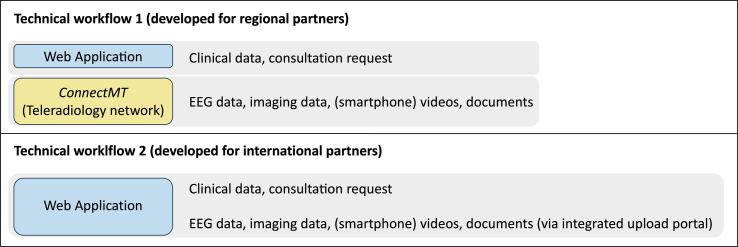
Intended workflows for regional partners (connected to the established teleradiology network) and international partners (with no access to the established teleradiology network).

As the technical requirements for the telemedical co-operation of the regional and international partners were not the same, different technical workflows were developed in each case, for overview see Fig. 2. Transfer of the clinical data and the request was carried out for both workflows by a web application programmed by Panvision GmbH. It is a browser-based single-page application that uses AngularJS in the front end and was developed on the server side with the.NET framework from Microsoft. Implemented mechanisms to ensure data security and data protection are the following:

• The web application is hosted on a secure server of Knappschaftkliniken Service GmbH
• Proof of identity for access to the web application is provided via 2-factor authentication (personal password and one time password)
• End-to-end encrypted data transfer
• Transfer of personal data for teleconsultation was only carried out after the patient had been informed in writing
• Privacy-by-design setting in the web application guaranteed that physicians actively confirmed that patient’s consent was existing before the data was transferred

The web application was not interoperable with the hospital information systems either at UKB or at any other study site, all data had to be entered or copied into the web application manually. The field formats used for the input mask (free text, selection fields with single or multiple selection, exact or open datum etc.) are described in Supplement 1.

**Fig. 3:**
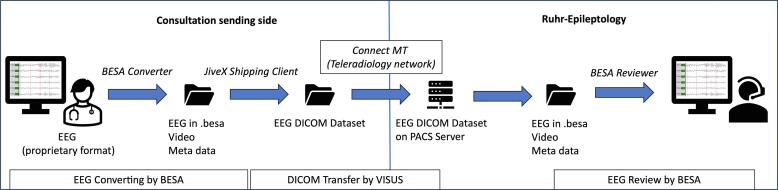
Transfer of the (video-) EEG via Teleradiology Network.

The technical workflow 1 (Fig. 2) with use of the established teleradiology network (ConnectMT operated by MedEcon Telemedizin GmbH) was developed for the regional arm of TE Ruhr. The EEG data was processed as described in Fig. 1 for type C solution (Converting EEG into a common format before EEG transfer), this ensured interoperability of the EEG data. Details of this process are demonstrated in Fig. 3. A converting software (*BESA Converter* programmed by BESA GmbH*)* was installed locally at the consultation sending site to convert video-EEG files from the proprietary EEG format to the BESA standard format “.besa” . The resulting three files (EEG in.besa format, video and *meta*-data) were stored in a folder. A software provided by VISUS GmbH (*JiveX shipping client*) stored these files in a ZIP archive in a DICOM RAW object. Additional files such as medical documents as PDF or JPEG and smartphone videos could also be added to the ZIP archive stored in the DICOM RAW object. This DICOM object was sent together with existing imaging data (DICOM studies) via the routinely used ConnectMT to Ruhr-Epileptology. Imaging data and EEG data were stored on the Picture Archiving and Communication Systems (PACS^6^) – Server. At Ruhr-Epileptology, the EEG data were extracted from the DICOM object. Then the video-EEG was examined using a BESA review software. MRI studies were read directly in the PACS. Table 3 summarizes the technical developments of the industrial partners.

**Table 3:** Developments of industrial partners.

EEG Converting by BESA GmbH	DICOM Transfer by VISUS GmbH
• Development of a standardized format for EEG data:.besa • Software development: BESA Converter	• Software development: JiveX shipping client • Implementation of the transfer via the teleradiology network ConnectMT

As the international partners did not have access to the regional teleradiology network, an upload portal was integrated into the web application which allowed the upload of EEG data, imaging data and other file formats (videos, doctors' letters, etc.) (Workflow 2 in Fig. 2). Regarding the EEG data, this worked as a type B solution described in Fig. 1 (EEG transfer and use of a multi data format reader). EEGs were reviewed at Ruhr-Epileptology in their proprietary format via BESA Research (BESA GmbH, Gräfeling) as a multi data reader. We use BESA Research in our department already for the presurgical work up, so there were no additional costs.

### 3.3 Implementation of TE Ruhrfor regional and international partners (primary endpoint)

Workflow 1 was established in two out of five cooperating departments in the regional arm and proved usable as intended. In the remaining three cooperating departments of the regional arm, the installation of the BESA-Converter as mandatory part proved to be impossible until 02/2020. Reasons were not systematically evaluated but the main problem appeared to be concerns of IT-departments regarding the installation of a foreign software (BESA Converter) and/or simply the lack of time to assist installing a solution for which the priority was not obvious neither to the IT-departments nor to the hospital managements.

Since workflow 2 was implemented in all three cooperating departments of the international arm rather easily, we decided in 03/2020 to stop the installation and further development of workflow 1 and instead implemented the workflow 2 also for our regional partner departments.

After establishing workflow 2 in 03/2020, all cooperating departments were able to use TE Ruhr. From 01/09/2018–30/06/2022 (46 months) 149 consults for 144 patients (age 4-85y) were completed. The departments of the regional arm initiated 69 consults (range 2–36, mean 8.6), the participants from the international arm 80 (range 6–68, mean 26.7).

The primary endpoint of TE Ruhr, the technical establishment of a tele-epileptologic platform, was thus achieved. At the same time, unexpected obstacles to the installation of telemedicine applications were recognized.

### 3.4 Evaluation of TE Ruhr by users

18 users from 8 participating departments of the regional arm participated in the online final evaluation survey and rated the project with an average score of 1.6 (1–3) on an established German school grading scale of 1–6 (1 − very good, 6 − very bad). The majority saw a benefit both for individual patient care and for improving their own epileptological expertise (mean 1.3 and 1.4, respectively, on a 5 point-Likert scale, (where 1 means complete agreement and 5 means complete disagreement).

Reasons given for the relatively infrequent use of TE Ruhr were:

• the time required for the consultation request was too high (n = 8, 44 %)
• the technical procedure was too complicated (n = 8, 44 %)
• specific questions rarely arose in everyday clinical practice (n = 7, 38 %)

(multiple answers possible).

Nevertheless, all respondents continued to see value in tele-epileptological consultation platform, thereof n = 8 (44 %) as a low-threshold routine tool and n = 10 (56 %) only for individual cases.

## 4 Discussion

TE Ruhr shows that the implementation of a comprehensive tele-epileptological consultation platform is technically feasible. All data formats that are needed to be transferred from the medical perspective, can be transferred. The collection of structured clinical data, in particular a detailed epileptological history enabled expert recommendations to be made by physicians of Ruhr-Epileptology even without direct contact with the patient. The physicians requesting consults also saw a benefit for improving their own epileptological expertise, which is certainly also due to training effects in the collection of the relevant clinical data.

### 4.1 Telemedical eeg data solutions

For the biggest challenge in TE, the handling of different EEG formats, TE Ruhr developed and tested two solutions:

• Converting EEG into a common format before EEG transfer in workflow 1 (cf. Fig. 1, solution C)
• EEG transfer and use of a multi data format reader in workflow 2 (cf. Fig. 1, solution B)

Workflow 2 had the clear advantage of being much easier to install and therefore prevailed in our project. The participating doctors only required internet access, access data for the web application and the ability to export examination data from the EEG system in order to subsequently upload them to the web application. In most cases, this required only minimal support from IT departments, so even physicians with less technology background were able to understand the process. The web application itself has adequate mechanism to ensure data security and data protection, which were approved by the Data Protection Officer and the Ethics Committee. Disadvantages were especially the limitations of the multi data format reader: due to missing comprehensive standard format, no synchronized video and EEG review was possible. Also, in many cases, EEG annotations were not included or not readable. Since these limitations impede the interpretation of (video-) EEG, they will have to be addressed. Workflow 1 used for the first time a software (BESA Converter) which allowed the conversion of EEG including video to a common format, with the hope of solving the limitations of the multi-format reader and ensuring interoperability for EEG data. The proof of technical functionality could be provided with two cooperating regional partner departments, yet due to the need to install additional software, this method was not able to establish itself as a commonly used technique in our study. The expressed wish of local doctors, including the department heads, was not able to overcome this obstracle. We assume that in settings that are characterized by more openness towards telemedicine solutions − possibly also because they are supported politically and financially − comparable blockades would have been less pronounced.

### 4.2 Lack of interoperability

Users indicated a high level of satisfaction with the system, but no dynamic of high patient or case throughput developed. Feedback from the users indicated that time and technical effort for the consultation request were the main reasons for this. In parts the complicated form design including the need to validate forms several times and to ask patients for consent were due to the requirements of a study. This certainly hindered integration of the tool into a daily routine. It can be expected that without a study-derived administrative overload, acceptance would have been higher. For clinical routine, an alternative to the technically complex EEG and imaging sharing could be a simple, web-based screening tool for epileptological questions without reference to patients by name, comparable to toolsforepilepsy.com (https://www.toolsforepilepsy.com). This may lead to a higher throughput in order to select patients who should be discussed in detail via telemedicine or referred in person at the epilepsy centre. However, for a comprehensive epileptological teleconsultation, a patient related case discussion and at least the evaluation of a standard EEG should be enabled.

With regard to usability of comprehensive epileptological teleconsultations, the lack of interoperability both for the clinical as the neurophysiological data, needs to be addressed. The web application enables clinical data to be transferred between regional and international partners but user-friendliness is limited due to the missing interaction with the primary system where most clinical data are already available digitally. Creating interoperability requires co-efforts from information system vendors to define and implement interfaces that are capable of exchanging semantically interoperable data. Regarding interoperability for neurophysiological data, efforts have been made for many years: Comparable to the.BESA-format, the European Data Format (EDF, EDF+) has been in use as a standard format for polygraphic data for many years *(*Kemp et al., 1992, Kemp and Olivan, 2003), several manufacturers provide it meanwhile (Alvarez-Estevez, 2024). However, Halford et al. pointed out some disadvantages of EDF/EDF+: lack of support for synchronized video, poor extensibility, lack of data compression, restrictions to 16 bit dynamic range, limited string length with no support for IEEE/ISO 11073 medical terminology, and lack of encryption for personal health information (Halford et al., 2021). Brinkmann et al published the multiscale electrophysiology format (MEF) for the first time in 2009 as a further development of the EDF (Brinkmann et al., 2009), Stead and Halford suggested in 2016 to use MEF as the standard format for neurophysiological data as some of the limitations of EDF have been resolved (Stead and Halford, 2016). In 2018, a taskforce was established in the International Federation of Clinical Neurophysiology (IFCN) to address the issue of standardization of neurophysiological data. This taskforce voted to proceed with DICOM as the standardization organization since the DICOM standard is already the worldwide communication standard in radiology. Also, it offers many advantages as a standard format for neurophysiological data. The DICOM Working Group 32 with support of the IFCN and partners in industry has created a standard for routine electroencephalography among others (polysomnography, electromyography, electrooculography) (Halford et al., 2021), this supplement enables Routine Scalp EEG’s been encoded as DICOM objects. Lang et al. recently showed that there is a great clinical use of the new DICOM Standard (Lang et al., 2023). This is to be considered the start for the adoption of EEG in an international standard. However, is does not meet all needs yet, e.g., a definition for video-EEG. Due to the long life cycles of EEG systems, it will take five to ten years before a relevant amount of DICOM ready EEG systems will have been installed. This would considerably simplify tele-epileptological collaboration and improve acceptance. Furthermore, it will enable the storage of EEG data in the already existing radiological archives (PACS). In the future, new concepts like secured cloud-based data access and backup will be implemented, also for neurophysiological data. Cloud computing is not yet widespread in the healthcare system, however, with Stratus EEG (Kvikna ehf., Reykavik, Iceland; Stratus, Irving, USA) there is now a provider that offers cloud-based EEG software (Stratus EEG, 2024) (cf. Fig. 1, type D solution).

### 4.3 Tele-epileptology: Heterogeneity of technical implementation

In the literature several other tele-epileptological networks are described, which shows the relevance of telecooperation, especially for complex variants of diseases with the need for specialized care and in rural regions. There is a great heterogeneity in the technical implementation of tele-epileptological solutions: In the Epilepsy Network Hessen Evaluation ENHE), a tele-epileptological project running in Germany in parallel to ours, the EEG data were transmitted in the original format in a DICOM RAW object via ConnectMT teleradiology network; a multi-reader was used for reporting (Type B solution). The detailed evaluation of ENHE also revealed that the usability of tele-epileptological consultations requires further improvement (Zöllner et al., 2024). Other national networks use remote access to report EEG data (personal communication), as is the case in a Spanish project between a secondary and a tertiary hospital (Campos et al., 2012) (Type A solution). In the UK, the central national health service (NHS) facilitates secure file transport systems between different hospital or provides shared servers, but even in this case, the EEG format and EEG reading software must be harmonized (Breen et al., 2010, Coates et al., 2012). Telemedical reporting of EEG in France is widely used for >10 years but transfer of EEG data is still not standardized *(*Sauleau et al. 2016).

## 5 Conclusion

In summary, proof was provided that a functioning telemedicine workflows could be established with a hospital-industry cooperation. The network structures established by TE Ruhr can be incorporated into standard care. Interoperability between hospital information systems and EEG solutions, as well as further standardization of neurophysiological data could improve acceptance in the future but at the same time deep integration into software requires cooperation with IT departments. Summarizing the experiences of our study, we understand that this mirrors the challenge of all telemedicine solutions, irrespective of their indication.

## Author Contributions

Sigrid Mues:

• Contributed in execution of the project and the analysis and interpretation of the data.
• Involved in drafting, writing and revising the manuscript.

Arndt Ebert:

• Contributed in technical developement.
• Involved in writing and revising the manuscript.

Marc Kämmerer:

• Contributed in technical developement.
• Involved in writing and revising the manuscript.

Marcus Kremers:

• Contributed in conception and the design of the project.
• Involved in revising the manuscript.

Ulrich Sliwka:

• Contributed in conception and the design of the project, recruitment of patients.
• Involved in revising the manuscript.

Rüdiger Hilker-Roggendorf:

• Contributed in conception and the design of the project, recruitment of patients.
• Involved in revising the manuscript.

Dirk Woitalla:

• Contributed in conception and the design of the project, recruitment of patients.
• Involved in revising the manuscript.

Iris Adelt:

• Contributed in conception and the design of the project, recruitment of patients.
• Involved in revising the manuscript.

Thomas Günnewig:

• Contributed in conception and the design of the project, recruitment of patients.
• Involved in revising the manuscript.

Anna Miron:

• Contributed in conception and the design of the project, recruitment of patients.
• Involved in revising the manuscript.

Sulev Haldre:

• Contributed in conception and the design of the project, recruitment of patients.
• Involved in revising the manuscript.

Kanjana Unnwongse:

• Contributed in recruitment of patients.
• Involved in revising the manuscript.

Wenke Grönheit:

• Contributed in conception and the design of the project.
• Involved in revising the manuscript.

Tim Wehner:

• Contributed in execution of the project.
• Involved in critically revising the manuscript.

Vanessa Behrens:

• Contributed in execution of the project.
• Involved in revising the manuscript.

Jörg Wellmer:

• Contributed in conception and the design of the project, its execution, and the analysis and interpretation of the data.
• Involved in drafting, writing and revising the manuscript.

All authors have read and proved the final version of the manuscript.

## Funding

This research was funded by a grant of Werner Richard – Dr. Carl Dörken Stiftung, Herdecke, Germany.

## Declaration of competing interest

The authors declare the following financial interests/personal relationships which may be considered as potential competing interests: [Arndt Ebert is employed by BESA GmbH. Marc Kämmerer ist employed by VISUS Health IT GmbH. Markus Kremers is employed by MedEcon Ruhr GmbH and VISUS Health IT GmbH].

## Footnotes

1 TE = tele-epilpetology, telemedicine in epileptology.

2 DICOM = digital imaging and communications in medicine.

3 GPDR = General Data Protection Regulation

4 UKB = University Hospital Knappschaftskrankenhaus Bochum.

5 TE Ruhr = Tele-Epileptology Ruhr

6 PACS = Picture Archiving and Communication Systems.
