# Supplementary material for: Multimodal tele-epileptology: Challenges on the way to interoperable medical data
Source: Clin Neurophysiol Pract. 2025 Feb 28;10:56–62. doi: 10.1016/j.cnp.2025.02.004 (PMC11927222; doi:10.1016/j.cnp.2025.02.004)
Supplement: Supplementary Data 1 [file mmc1.pdf]

General information on epilepsy / illness

A diagnosis of "epilepsy" is	○ Confirmed ○ Likely ○ Unlikely
Previous assignment of the epilepsy type	*
Previous aetiological assignment of epilepsy	**
Relevant comorbidity	2500 characters remaining
If no epilepsy, what is the (suspected) diagnosis?	***
Further information of alternative diagnoses	2500 characters remaining
Since when do seizures occur?	○ Date ○ Free entry
If epilepsy: the etiology is	○ Known ○ Suspected ○ Unknown

*
- focal epilepsy
- Generalized epilepsy
- Combined focal and generalized epilepsy
- not clear

**
- Structurally
- Genetically
- Infectious
- Metabolically
- Immunologically
- Not clear

***
- Psychogenic non-epileptic seizures only
- (Suspected) psychogenic and epileptic seizures
- Syncopes
- Syncopes + epileptic seizures
- Other

Previous findings / extended anamnesis

**Seizure semiology**	2500 characters remaining
**Results of previous EEG**	2500 characters remaining
**Epileptiform activity/seizures recorded**	○ yes ○ no
**MRI head available**	○ yes ○ no
**Abnormalities in early childhood development**	○ yes ○ no
**Epilepsy Predisposing Factors**	☐ Febrile seizure ☐ TBI ☐ Inflammatory CNS disease ☐ Ischemia ☐ Tumour ☐ OP ☐ Others
**Abnormalities in neurological findings**	2500 characters remaining
**Family history**	
**for epilepsy**	○ positive ○ negative
**for other diseases**	○ positive ○ negative
**Is there currently / foreseeable family planning**	○ yes ○ no

Medications

Current anticonvulsive medication(s)

Drug name	Dosage (mg/day)	Dosage since when?	Since when, maximum dosage, side effects (free text)
*	0		

+ further substance

Current additional medication

2500 characters remaining

Comorbidities

2500 characters remaining

Previous anticonvulsive drugs

+ further substance

Side effects, which anticonvulsants were particularly effective, other peculiarities

2500 characters remaining

*

PB (Phenobarbital)	BRV (Brivaracetam)	ST (Sultiam)
PRM (Primidon)	TPM (Topiramat)	STP (Stiripentol)
PHT (Phenytoin)	ZNS (Zonisamid)	CLB (Clobazam)
CBZ (Carbamazepin)	PGB (Pregabalin)	DZM (Diazepam)
OXC (Oxcarbazepin)	GBP (Gabapentin)	LZM (Lorazepam)
ESL (Eslicarbazepin)	PER (Perampanel)	CZM (Clonazepam)
LTG (Lamotrigin)	VPA (Valproat)	PZM (Pirazetam)
LCM (Lacosamid)	ESM (Ethosuximid)	RTG (Retigabin)
LEV (Levetiracetam)	MSM (Mesuximid)	

Why is the patient currently admitted to your department

- [ ] First seizure
- [ ] Single, but not first seizure
- [ ] Series of seizures
- [ ] Status epilepticus
- [ ] Unsatisfactory seizure control
- [ ] Seizure freedom - need to continue medication?
- [ ] Differential diagnosis epileptic vs. non-epileptic seizures
- [ ] Search for cause of epilepsy
- [ ] Side effects of medication
- [ ] Special situations / comborbidities
    - [ ] Pregnancy
    - [ ] Dysphagia
    - [ ] Renal insufficiency/dialysis
    - [ ] Hepatic insufficiency
    - [ ] Suspected side effects of medication
    - [ ] Planned surgery
    - [ ] Palliative situation
    - [ ] Other
- [ ] Other reason for the consultation

Request to consulting institution

- [ ] Request for diagnostic confirmation
- [ ] Suggestion for further diagnostic work-up
    - [ ] Diagnostic imaging
    - [ ] EEG Diagnostics
    - [ ] Neuropsychological testing
    - [ ] Speech lateralisation and localisation diagnostics
    - [ ] Genetic diagnostics
    - [ ] Immunological diagnostics
    - [ ] Metabolic diagnostics
- [ ] Special additional examinations
    - [ ] MRI co-diagnosis
    - [ ] MRI-postprocessing (MAP18)
    - [ ] EEG co-diagnosis, routine-EEG
    - [ ] EEG co-diagnosis, video EEG Monitoring
    - [ ] EEG source localization
    - [ ] Co-diagnosis neuropsychological testing
- [ ] Presurgical work-up indicated?
- [ ] Request for therapeutic recommendation
    - [ ] Acute treatment of epilepsy (emergency)
    - [ ] Acute treatment of status epilepticus (emergency)
    - [ ] Urgent treatment of epilepsy
    - [ ] Elective treamtent advice
- [ ] Request for admission to Ruhr-Epileptology
- [ ] Request for epilepsy-specific counselling (social/vocational aspects)

Request to consulting institution (free entry)

2500 characters remaining

Examination files

Upload | File formats | Protocol | Zip | Download | Download (ZIP) | Rename | Delete

teust, nathanel 10570

Name	Type	Change date	Size
